# Exploring the role of community engagement in improving the health of disadvantaged populations: a systematic review

**DOI:** 10.3402/gha.v8.29842

**Published:** 2015-12-18

**Authors:** Sheila Cyril, Ben J. Smith, Alphia Possamai-Inesedy, Andre M. N. Renzaho

**Affiliations:** 1 Department of Epidemiology and Preventive Medicine, School of Public Health and Preventive Medicine, Monash University, Melbourne, VIC, Australia; 2 School of Social Sciences and Psychology, Western Sydney University, Penrith, NSW, Australia; 3 Department of Epidemiology and Preventive Medicine, School of Public Health and Preventive Medicine, Monash University, Melbourne, VIC, Australia; 4 Office of the Pro-Vice Chancellor Arts (Education), Western Sydney University, Bankstown, NSW, Australia; 5 Humanitarian and Development Studies, School of Social Sciences and Psychology, Western Sydney University, Penrith, NSW, Australia

**Keywords:** community engagement, health, disadvantaged populations, ethnic minorities, culturally and linguistically diverse

## Abstract

**Background:**

Although community engagement (CE) is widely used in health promotion, components of CE models associated with improved health are poorly understood. This study aimed to examine the magnitude of the impact of CE on health and health inequalities among disadvantaged populations, which methodological approaches maximise the effectiveness of CE, and components of CE that are acceptable, feasible, and effective when used among disadvantaged populations.

**Design:**

The systematic review followed the Preferred Reporting Items for Systematic Reviews and Meta-Analyses guidelines. We carried out methodological assessments of the included studies using rating scales. The analysis focussed on model synthesis to identify the key CE components linked to positive study outcomes and comparative analysis between positive study outcomes, processes, and quality indicators of CE.

**Results:**

Out of 24 studies that met our inclusion criteria, 21 (87.5%) had positively impacted health behaviours, public health planning, health service access, health literacy, and a range of health outcomes. More than half of the studies (58%) were of good quality, whereas 71% and 42% of studies showed good community involvement in research and achieved high levels of CE, respectively. Key CE components that affected health outcomes included real power-sharing, collaborative partnerships, bidirectional learning, incorporating the voice and agency of beneficiary communities in research protocol, and using bicultural health workers for intervention delivery.

**Conclusions:**

The findings suggest that CE models can lead to improved health and health behaviours among disadvantaged populations if designed properly and implemented through effective community consultation and participation. We also found several gaps in the current measurement of CE in health intervention studies, which suggests the importance of developing innovative approaches to measure CE impact on health outcomes in a more rigorous way.

Over the past few decades, community engagement (CE) has emerged as an increasingly effective strategy for harnessing community potential, particularly in health improvement (1). CE has been widely used by health interventionists to engage communities in health promotion, research, and policy making to address health issues including obesity, cancer, heart disease, diabetes, and mental illness (2–4). CE is defined as ‘a process of working collaboratively with groups of people who are affiliated by geographic proximity, special interests, or similar situations, with respect to issues affecting their well-being’ (5, p. 9).

There are several CE models being used in health studies, including the Social Ecological model, the Active Community Engagement Continuum, Diffusion of Innovations, and community-based participatory research (CBPR) (6), that aim to initiate population-level changes in health through the active involvement of the community. CBPR is often used synonymously with participatory action research (PAR) and action research, which include participatory approaches to health research (7, 8). In contrast to the other CE models, CBPR has sought to bridge the gap between research and practice through equitable engagement of the community to eliminate disparities in population health (9). CBPR has achieved this by addressing power imbalances and enabling knowledge exchange, resulting in its wide uptake as an appealing CE approach across various cross-cultural, diverse, and disadvantaged settings (9, 10). Additionally, Rapid Assessment Response and Evaluation (RARE), a component of PAR, has emerged as a valuable public health research tool, particularly among ethnic populations, and incorporates the use of datasets, community participation, and evaluation (11). Staley's (12) comprehensive review identified key areas where CE can positively impact health research, including agenda setting, ethical conduct, programme design and delivery, involvement of the public in a project, and academic partnerships.

CE has also been advocated as a tool for providing a ‘voice to the voiceless’ and therefore is considered valuable for tackling health inequalities (13). Disadvantaged groups often experience health inequalities and bear a disproportionate burden of disease as a result of structural, social, and cultural barriers (8, 14, 15). Disadvantaged populations are challenged by geographic access to healthcare, culturally inappropriate services, financial barriers, poor health literacy, and language barriers (16–18), which impede their effective utilisation of health services. Additionally, they often have higher risk factors for diseases, lack of awareness of the existing health resources, and poor eligibility for health insurance, further limiting their access to healthcare (15, 19, 20). However, health interventionists tend to use CE approaches that have worked among non-disadvantaged populations for disadvantaged groups, often resulting in failure to achieve the desired outcomes (21–23). Current evidence shows that disadvantaged populations are not adequately approached or effectively engaged in the efforts taken by service providers and health interventionists to improve their health (24–26).

Furthermore, Wallerstein (27) reported that disadvantaged populations are also disempowered and unable to engage in traditional health promotion programmes in which individuals are encouraged to take control over their health. Importantly, in developed countries, people from non-English speaking backgrounds are often under-represented in population health studies, thereby excluding them from health promotion policy and programmes, resulting in unmet social and health needs (28). Additionally, the exclusion of disadvantaged groups from public health policy initiatives has the potential to widen health disparities (29). Hence, there is an urgent need to develop CE initiatives that align with the community's cultural framework, to improve the social inclusion of marginalised people (30), improve research quality, and address health disparities (31).

## Current evidence gaps

The existing literature shows that there is a lack of consistency in the effectiveness of CE in improving the health of disadvantaged populations. Although some studies have found that CE did not improve health behaviours (32–35) or health outcomes (36, 37) among disadvantaged populations, others have reported that CE had positive impacts on health behaviours (38–40) and health outcomes (41, 42) among these groups. Popay et al. (43) found that although CE improved social capital, cohesion, and empowerment among disadvantaged populations, it did not have any positive impact on mortality, morbidity, health behaviours, or health inequalities. Despite its potential for empowering disadvantaged populations, studies have shown that the majority of the health programmes use ‘top-down’ CE approaches, as opposed to ‘bottom-up’ participatory methods, which limit their impact upon health and health behaviours (44, 45).

Adding to the complexity of this literature, Attree et al. (46) found that despite achieving benefits in physical health, CE may result in unintended negative consequences such as exhaustion, financial burden, consultation fatigue, and disappointment for some participants, who were repeatedly exposed to successive waves of CE, which adversely affected their health. Additionally, those with disabilities found the physical process of engagement extremely difficult, since their special needs were not considered during the planning of CE meetings (46). Chau (47) found that using payments as engagement incentives had negative consequences such as bullying of and discrimination against ethnic community members by other participants, resulting in the breakdown of trust in the engagement process. These studies mostly relied on consultation as the prime process of engagement without giving ownership to the community, resulting in negative engagement experiences for the participants (46–48). O'Mara-Eves et al. (45) showed that although public health interventions that include CE appear to be effective across populations and contexts, the evidence is less clear about how CE should be implemented to maximise impact on the desired outcomes for disadvantaged populations. Overall, there is a lack of conclusive evidence on the role of CE in improving the health of disadvantaged populations and whether the identified improvements in health are due to the intervention itself, the CE approach, or both (45, 48).

We conducted a systematic review to address these gaps by examining the magnitude of the impact of CE on health and health inequalities among disadvantaged populations. We set out to investigate which methodological approaches maximise the effectiveness of CE and which components of CE are acceptable, feasible, and effective when used among disadvantaged populations.

## Methods

### Protocol

We conducted the systematic review according to the Preferred Reporting Items for Systematic reviews and Meta-Analyses (PRISMA) guidelines (49).

### Information sources

We conducted a comprehensive search of both peer- and non-peer-reviewed articles from computerized bibliographic databases using relevant MeSH words or subheadings of key words as outlined in Table 1. The search was limited to articles published in English and during the period January 1995 to June 2015. We imported the articles retrieved from each of the databases into an Endnote library.

**Table 1:** Search strategy

Database	Search strategy
MEDLINE, CINAHL, PsycINFO, IBSS, PubMed, Google Scholar, ArticlesPlus, Scopus, Sociological Abstracts, Web of Science, ProQuest, the Campbell Collaboration Library of Systematic Reviews, the Cochrane Library, OneFile (Gale), Academic OneFile, PAIS International, Australia Policy Online, WorldWideScience.org, and Embase	Community engagement, or community networks, community-institutional relations, community-based participatory research, consumer participation, or community control AND Disadvantaged population, or healthcare disparities, socioeconomic factors, health services accessibility, ethnic groups, ethnic communities, population dynamics, marginalised populations, sexual minorities, refugees, ‘transients and migrants’, vulnerable populations, homeless persons, mental disorders, mental illness, disabled or disabled persons AND Health, or health services, health knowledge attitudes practice, health literacy, health policy, health facilities, health planning, health behaviour, health education, health status disparities, health promotion, evaluation studies, feasibility studies, programme evaluation, validation studies, randomized controlled trials, or intervention studies

### Inclusion and exclusion criteria

We included studies in the review if they met the following criteria: a) described the role of CE in health intervention studies among disadvantaged populations; b) used CE to develop health programmes for disadvantaged populations; c) evaluated CE as an intervention component; and d) were published between January 1995 and June 2015.

We excluded studies if they a) focussed solely on the development of CE models without studying their impact on the health of disadvantaged populations; b) did not clearly describe the CE model they used; or c) were letters, opinion pieces, review articles, or theses.

Disadvantaged populations included those of low socio-economic status, ethnic minorities, sexual minorities, culturally diverse populations, indigenous groups, and marginalised groups such as people with disabilities and the homeless.

### Data extraction and synthesis

Data extraction followed a three-step process with articles filtered by title, abstract, and full text. One of the authors (SC) initially screened the potentially relevant studies. Three authors (SC, BJS, and API) independently reviewed articles retained for inclusion in the preliminary phase. Similar to a process used by Renzaho et al. (50), we extracted data on the characteristics of included studies, including study research design, population under study, setting, sample size, study results, and limitations. Data on the type of CE models used in each study, the CE model components, the impact of CE on study outcomes, the extent of involvement of CE partners in the research study activities, and the level of CE achieved by the particular model were extracted using a piloted form. To pilot the abstraction process, two researchers (SC and AR) independently reviewed 10 randomly selected papers and compared the results in face-to-face meetings to ensure that a consistent approach was taken to evaluate the selection criteria.

We conducted model analysis to analyse the processes used in each CE model and identify the key CE components directly contributing to positive study outcomes. Following this step, we carried out comparative analysis between positive study outcomes, CE processes, and quality indicators. Finally, we synthesised data on the mechanisms of CE and their relationship to study outcomes, on the key similarities and differences between the various CE models, and on the distinguishing features between CE approaches achieving positive versus negative impacts on study outcomes.

### Quality assessment

Quality assessments of the included studies were conducted in three phases independently by two authors (AR and SC) using the appropriate scoring instruments and rating scales described in each phase, with consensus reached through discussion and comparison of scoring sheets. Phase 1 focussed on the methodological approaches used by the individual studies. For assessment of research quality in Phase 1, CONSORT criteria (51) were used to assess randomised controlled trials (RCTs), the STROBE checklist (52) was used for longitudinal studies, the McMasters qualitative review tool (version 2.0) (53) was used for qualitative studies, and mixed methods studies were assessed using the Evaluative Tool for Mixed Method Studies (54). Scoring sheets were developed to assess the research methodology and adherence to scoring tool criteria for each included article; studies scoring <30% against the criteria were classified as poor, those scoring between 30 and 70% were classified as moderate, and those scoring >70% were classified as good quality studies. The results of the Phase 1 methodological assessment of research studies are summarised in Supplementary Table 1.

In Phase 2, quality assessments of the CE models were carried out using rating scales that examined the level of engagement achieved and the extent of engagees’ involvement in research. First, based on the IAP2 Public Participation Spectrum (55), the level of engagement was measured across five levels as follows: 1) *informing* – providing the community with information on the programme; 2) *consulting* – listening to community feedback, not allowing new ideas; 3) *involving* – allowing joint decision-making; 4) *collaborating* – forming a partnership to carry out the decisions; and 5) *empowering* – placing final decision-making in the hands of the community. Studies were rated using a scoring tool (Levels 1–2=poor; Levels 3–4=moderate; Level 5=good).

Second, guided by the framework developed by Rifkin et al. (56) and the CBPR principles developed by Israel et al. (57), we assessed the extent of involvement of engagees (community involvement) by using evidence of their participation in the following aspects of the research study as criteria: 1) needs assessment; 2) design and development of the programme; 3) recruitment and retention of participants; 4) development of study instruments; 5) implementation of intervention; and 6) data interpretation, analysis, and dissemination of results to the community. Studies that fulfilled two or fewer criteria were classified as having poor community involvement; studies fulfilling three to four criteria were classified as having moderate community involvement; and studies fulfilling five or more criteria were found to have good community involvement.

In Phase 3, the relationship between the CBPR model components and study outcomes was analysed using the conceptual logic model, which comprises four dimensions: context, group dynamics (including structural, relational, and individual sub-dynamics), intervention, and outcomes (9, 58). The results of the quality assessments conducted in Phases 2 and 3 are summarised in Supplementary Table 2.

## Results

**Fig. 1:**
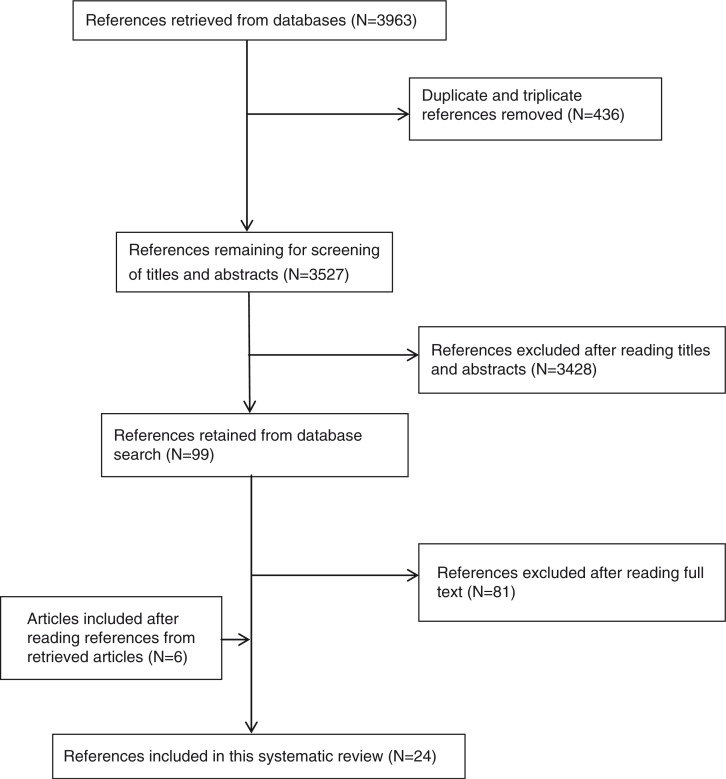
Flow chart of study selection.

The search resulted in 3,963 articles; following removal of duplicates 3,527 articles remained, out of which 3,428 articles were excluded after screening the titles and abstracts. The remaining 99 articles were read and checked for eligibility, leading to 81 articles being excluded. Eighteen articles met the inclusion criteria, and a manual search of the bibliographic lists of the 18 articles led to the inclusion of six additional articles, giving a total of 24 studies (Fig. 1).

### Characteristics of the included studies

Of the 24 studies included in this review, 17 were conducted in the United States, and there was one each in Canada, Bangladesh, Africa, China, the United Kingdom, Iran, and India. The studies used various designs, including RCTs (*n*=11), quasi-experimental (*n*=2), longitudinal (*n*=2), qualitative (*n*=4), and mixed methods (*n*=5). The sample size varied from 23 to 3,986, with study populations having a variety of ethnic backgrounds, including African American, Hispanic, Indian, African, Chinese, Iranian, and indigenous First Nations communities. Thirteen studies focussed on the improvement of health behaviours, four studies examined maternal/neonatal health outcomes, two studies focussed on breast cancer, two studies examined mental health, one study examined sexual health among homosexual men, one study examined childhood asthma, and one study was on influenza pandemic planning. Out of 24 studies that met our inclusion criteria, 21 (87.5%) reported improvements in health behaviours, public health planning, health service access, health literacy, and a range of health outcomes (Supplementary Table 1). Fourteen studies were found to be of good quality (58%), six studies (25%) were moderate in quality, and four studies (17%) were poor in quality (Supplementary Table 1).

### Analysis of the CE models

We identified 11 categories of CE initiatives in this review, including CBPR, which was used in 12 (50%) studies, and community-partnered participatory research (CPPR), a variant of CBPR, which was used in two studies. The other nine categories include the community health worker (CHW) model, community empowerment model, community action cycle, youth development model, the Well London model, participatory action cycle, the FOCUS (Families in Our Community United for Success) model, the Culturally appropriate Diffusion Communication (CDC) model, and ANGELO (Analysis Grid for Elements Linked to Obesity), all of which were used in one study each. Fourteen studies (58%) in this review showed that CE-informed research led to reductions in health inequalities, by showing improvements in health behaviour and outcomes among disadvantaged populations bearing a disproportionate burden of disease compared to the mainstream populations (3, 4, 18, 59–69).

Level of CE achieved was moderate in 15 studies (63%), out of which eight studies used CBPR (3, 24, 60–62, 67, 70, 71), two studies used the CHW model (59, 63), and the remaining five studies used one of the following models: the FOCUS model (72), ANGELO model (73), the community empowerment model (66), the participatory action cycle (65), and CPPR (4). The level of CE achieved was good in six studies (25%), four of which used CBPR (18, 68, 69, 74), one used CPPR (75), and one used the CDC model (64).Three studies (12%) that used the community action cycle (76), youth development model (77), and Well London model (78) showed poor levels of CE, where the community was only informed or consulted. The extent of involvement of engagees in the research project was good in 17 studies (71%), out of which 12 were CBPR studies (3, 18, 24, 60–62, 67–71, 74), two were CPPR studies (4, 75), and three studies used the community empowerment model (66), the CDC model (64), and the CHW model (59). Four studies (17%) that used the FOCUS model (72), participatory action cycle (65), the ANGELO model (73), and the community action cycle (76) showed moderate community involvement, while three studies (12%) that used the CHW model (63), the Well London model (78), and the youth development model (77) showed poor community involvement.

Twelve studies (50%) evaluated the CE-informed intervention, out of which eight studies conducted process evaluations (3, 18, 62, 68, 70–72, 78); three studies conducted formative, process, and outcome evaluations (59, 66, 67); and one study conducted an outcome evaluation only (74). Areas of CE commonalities in terms of effectiveness include conducting the research within the context of health issues of high perceived severity (3, 4, 18, 59, 60, 62, 65, 66, 69, 71, 76) and conducting needs assessments to identify barriers specific to each community (3, 4, 18, 59, 60, 62, 65, 66, 69, 71, 76) (Supplementary Table 2). Despite several studies using CE to enable the cultural adaptation of survey instruments and the cultural appropriateness of the programme (4, 18, 59, 60, 62, 68, 69), only one study clearly demonstrated the cultural validity of the adapted programme (59).

Factors facilitating the effectiveness of CE models included partner input in intervention design (4, 60, 61, 67, 69), shared learning between academic and community partners, and bridging people on research teams (3, 4, 61, 62, 75). On the other hand, poor community involvement in the design of survey instruments (77), lack of understanding of the communities’ knowledge of health issues, lack of bidirectional translation and implementation (76, 77), and failure of advisory councils to adequately motivate study participants and CHWs (76, 77) were the key factors responsible for poor study outcomes. Further, only two studies were able to demonstrate the positive impact of long-standing CE partnerships on study outcomes (67, 69). Regarding the sustainable impacts of CE, seven studies were able to show that CBPR led to empowerment as a result of the capacity of advisory councils and community voices being heard (3, 18, 24, 62, 69, 71, 75), whereas six studies reported policy change as a CBPR outcome (18, 24, 68–70, 74).

The majority of the studies (62%) used CBPR and CPPR to develop and implement the intervention, enable participant recruitment, coordinate data collection strategies, assist in the interpretation of results, and facilitate dissemination of findings (3, 24, 59–61, 69–71, 74, 75). Evaluation of the CE processes used in the included articles showed that the following were the most frequently identified elements of programme success: a) establishment of community advisory councils (3, 24, 60, 65, 68, 71, 73, 74) and collaborative partnerships (4, 18, 24, 62, 66, 68–70, 77) involving accountability of stakeholders towards all project activities; b) real power-sharing between the community and research team including bidirectional learning (3, 18, 24, 59, 61, 62, 68, 71, 75); c) formative research for programme development and mobilisation of appropriate community resources (3, 59, 67, 70–73); d) community involvement in research design and integration of culturally competent elements with the programme, including translations (3, 18, 24, 59, 62, 64, 73); e) training and ongoing support of bicultural CHWs (3, 59, 62, 63, 67); and f) incorporating the voice and agency of indigenous and ethnic communities in the research protocol (3, 18, 24, 59, 62, 66, 71–73).

### Impact of CE on study outcomes

The results for each of the 24 included articles are presented in Supplementary Table 2. Eight studies reported positive impacts of CE on health behaviours, including healthy eating (3, 59), physical activity (59, 60, 70, 71), breastfeeding (66), and condom use (64). Balcázar et al. (3) and Bender et al. (59) showed that using CHWs led to improvement in health behaviours by ensuring the cultural adaptability and acceptability of the programme, and Pazoki et al. (60) and Cohen et al. (70) found that CBPR-facilitated needs assessment identified community-specific barriers to physical activity, which were subsequently addressed in the programme design. Similarly, the community empowerment model used by Wright et al. (66) was found to improve breastfeeding practices among Navajo tribal women by identifying tribal barriers to breastfeeding and misconceptions instigated by marketing materials from formula feed companies. Gao and Wang (64) stated that the CDC model resulted in improved condom use through participatory communication approaches such as bar-based edutainment for gay priority groups.

One-quarter (*n*=6) of the studies reported positive impacts of CE on health outcomes, including reduction in obesity (69, 71), improvement in mental well-being and quality of life (4, 62), and reduction in neonatal mortality (63, 65). Chomitz et al. (69) showed that a history of collaboration and trust between the leadership and the community achieved reduction in obesity among ethnically diverse children, whereas Kim et al. (71) found that the lay health advisor model achieved obesity reduction among rural African Americans in a faith-based weight loss programme through goal-setting, faith orientation, and the use of community resources. Using traditional birth attendants as CHWs to deliver home-based neonatal care reduced neonatal mortality among communities with low health service utilisation in Bangladesh (63), whereas the participatory action cycle model reduced neonatal mortality among tribal women in India by identifying barriers to safe delivery practices and improving literacy concerning hygienic delivery and post-partum care (65). Wells et al. (4) reported that the bidirectional knowledge exchange used in CPPR enabled the improvement of mental well-being among depressed adults, while Nápoles et al. (62) showed that using CBPR-based formative research and peer facilitators to deliver the intervention improved the mental quality of life among breast cancer survivors.

Three studies reported increased awareness and improved knowledge of health issues among participants (60, 64, 72), and two studies also demonstrated improved participation in health screening programmes (18, 67). The FOCUS model resulted in increased awareness of teen pregnancy through community dialogue and revitalisation at a community kick-off breakfast, development of tailored posters on teen pregnancy prevention, and prioritisation of youth needs (72). Harvey et al. (67) showed that CBPR-facilitated formative research contributed to the training of CHWs, which improved hypertension screening among ethnic women with low health service utilisation. Similarly, English et al. (18) found that CBPR collaborative partnerships with tribal Navajo Indians enabled the cultural adaptation of the intervention, which improved their participation in mammography programmes.

Four studies reported community-level changes, including improvements in community empowerment (68), community-level health initiatives (74), public health planning (24), and the use of public parks (70). Collie-Akers et al. (74) reported that CBPR-facilitated collaborative partnerships led to policy changes, environmental improvements, and new health-related translation services in the community. Further, Charania et al. (24) stated that CBPR-driven advisory councils enabled important community-specific modifications to the original influenza pandemic plan, such as surveillance information, health services, supplies, drugs, and infection control protocol lists among First Nations communities living in sub-Arctic Canada. Ferrera et al. (68) showed that the non-hierarchical approach in their CBPR-driven programme resulted in social capital and empowerment among immigrant youth. Three studies that used the youth development model, Well London model, and the community action cycle reported no impact of CE on health behaviours such as smoking (77), healthy eating, and physical activity (78) or on utilisation of family planning services (76), respectively.

## Discussion

The findings from this systematic review showed that the CE approaches in 21 out of 24 studies that met our inclusion criteria led to improvements in health behaviours, public health planning, health service access, health literacy, and other health outcomes. Our findings of CE reducing health inequalities in 60% of the included studies are consistent with the current evidence (45, 46, 79) but not supported by other studies (76–78). For CE interventions that had positive impacts, components closely associated with improved health and health behaviours included incorporating the voice and agency of indigenous and ethnic communities in the research protocol, real power-sharing, bidirectional learning, and needs assessment. In contrast, CE models that did not improve health behaviours were affected by lack of community involvement in formative research and inadequate needs assessment. This is consistent with the findings of Israel et al. (57), who state that adequate community involvement is imperative towards achieving community change. In terms of health service utilisation, CE components such as tribal agency partnerships and cultural adaptation of programmes were instrumental in achieving improved outcomes.

We have identified CBPR as the most commonly used CE model, which is consistent with the current literature citing CBPR as the most successful approach for engaging ethnic and racial minority populations in health research studies (10, 80). Contrary to reports that CBPR has been effective only in achieving high retention rates and not in data analysis, interpretation, or dissemination (10), we found six studies that involved community partners in these stages of the research process as well. Apart from CBPR, we identified six other CE models that have successfully addressed health disparities among disadvantaged populations: FOCUS, ANGELO, CDC, community empowerment, the CHW model, and participatory action cycle. Although most of these models share similarities with the CBPR model, they lack three components that were key drivers of success in the CBPR model. These were engagement of community partners in all stages of research development including dissemination of findings, facilitating knowledge exchange between community and academic partners, and achieving balance between research and action.

### CE model components impacting study outcomes

The available literature on CE states that there is currently an evidence gap in understanding which CE components contribute to successful study outcomes (45, 48). This review examined CE levels along a continuum from informing communities to empowering them and found a link between low levels of CE (information-sharing and consultation) and poor study outcomes in three studies (76–78). On the other hand, studies achieving high levels of CE such as collaboration, partnerships, and empowerment showed positive study outcomes. A number of studies have found that CHWs can be successful in addressing health disparities among ethnic populations (81–83). Our review showed that using CHWs among ethnic communities improved programme feasibility and impact by enhancing the relevance of health promotion messages, fostering improved health behaviours, overcoming cultural and access barriers, and encouraging participant engagement. O'Mara-Eves et al. (45) showed that the ongoing training of CHWs and the quality of relationships between them and the participants affected study outcomes. Several studies in our review used a combination of CE approaches such as CBPR to develop collaborative partnerships and CHWs to deliver health interventions. Adopting such an approach had a two-fold benefit, where the community partners facilitated recruitment and training of CHWs, while CHWs accessed ‘hard to reach’ participants experiencing health disadvantages and enabled their retention.

Another CE indicator of study success found in our review was collaborative partnerships, which facilitated an improved understanding of traditional tribal and ethnic health beliefs among academic and other partners, enabling the development of locally relevant health policy initiatives for these groups. Similar to South and Phillips (84), we found that a range of CE tools such as surveys, forums, and photovoice enabled the establishment of these partnerships. Our review showed that new partnerships between community, government, and academic stakeholders and the use of existing infrastructure such as faith networks, park authorities, and tribal agencies were responsible for the post-intervention sustainability of programmes. Studies conducted among ethnic and tribal communities have shown that post-programme intervention effects were directly related to their cultural acceptability, the existence of a historical collaborative partnership, and the engagement of an influential community partner such as a government organisation or tribal agency in all stages of the research (3, 18, 24, 66, 69).

Some of the model-specific success indicators included the prioritisation process used by the ANGELO model, which enabled ‘community validation’, an important engagement factor in collectivist cultures (73), and the asset-building process used by the FOCUS model to improve health literacy among ethnic communities. South and Phillips (84) stated that assets within a community are considered the building blocks for community health. Participatory health communication strategies were used by the CDC model to enable penetration into socially marginalised groups, and ‘collective agency’ with local tribal agencies was a feature of the community empowerment model, which influenced positive breastfeeding behaviours. The participatory action cycle model addressed critical consciousness, which in turn empowered communities to take control over their health and other difficulties arising from poverty.

Further, we found that identifying needs unique to each ethnic community during the formative research phase was directly responsible for positive outcomes. Examples of these needs among culturally diverse and tribal communities include lack of childcare (59), traditional barriers to hygienic birth practices (65), barriers to health information access among homosexual men (64), fear of dying (62), fear of talking about cancer (18), and traditional beliefs preventing healthcare utilisation (65). The bidirectional translation and uptake of cultural concepts not only enabled community-specific needs to be identified and addressed in the intervention design, but were also responsible for programme satisfaction and retention. Overall, CE models identified in this review employed collaborative partnerships, bicultural CHWs, community participation, and power-sharing as key components of health interventions, a finding consistent with current evidence (45, 57, 80).

### Non-health impacts of CE

Our systematic review has identified several non-health-related positive outcomes of CE, such as building of social capital, community capacity building, and empowerment of community members leading to community championship, which are similar to the findings reported by Popay et al. (43). We found that CBPR enabled external partner organisations to achieve their goals by facilitating trust-building between native and academic communities. Our findings on CE facilitating referrals to social services, increasing the quality of local services, and enabling linkages with community resources were supported by the findings shown by Milton et al. (79). Other positive impacts of CE include identification of homelessness among depressed study participants and establishment of community-based health homes. Contrary to the evidence stating that CE participants experience emotional distress and stress (46), our findings suggest that the majority of CE participants were empowered and improved their social networking and self-efficacy skills.

### Challenges associated with implementing CE models and their uptake

Six of the included CBPR studies (3, 4, 60–62, 70) reported a trade-off between tailoring the intervention to suit the community's needs and the rigorous standardisation required in RCTs. The iterative dialogue with the community resulted in mid-programme adaptations, affecting the rigour of the RCT. Jensen et al. (85) found that high-quality CE was accompanied by a trade-off in research scientific methods, which was echoed by Balcázar et al. (3), who showed that dissemination of baseline results to the entire community improved community participation but also led to control group contamination that compromised the evaluation of their intervention. Sanson-Fisher et al. (86) have argued that due to a number of factors, including time for follow-up, external validity, and contamination of control groups, CE interventions cannot be effectively evaluated using RCTs. Others have stated that instead of viewing intervention fidelity and community needs-based adaptability as mutually exclusive factors, health interventionists should bridge the gap by integrating them within interventions and allow for some level of flexibility in peripheral community elements (87, 88). Rifkin (89) remarked that measuring community participation indicators and collecting a wider range of data on CE processes will enable a more holistic analysis of CE studies rather than the traditional RCT approach, which may not be capable of illustrating community changes in a comprehensive manner.

Although most studies included in our systematic review adopted CBPR approaches, only a few actually achieved high levels of CE, such as community control and empowerment, due to funding constraints and insufficient capacity of social and welfare services to address community needs. Swainston and Summerbell (48) found that power struggles between stakeholders and lack of funding and infrastructure were key barriers to CE. Uneven receptiveness from the community, lack of goal-sharing among stakeholders, community mobility, and differing priorities among advisory councils on resource-spending were some of the other reasons for non-effective CE. According to Israel et al. (90), having the ‘right people around the table’, a combination of structure and flexibility in rules governing partnerships, and adherence to CBPR principles in collaborations can be effective strategies for overcoming these challenges.

Implementing CE among disadvantaged communities has highlighted complex challenges, including poor health system infrastructure and service delivery, poor staffing and resources, and limited access to health services. These challenges often result in unmet community needs, causing community partners and study participants to feel dispirited, thereby compromising the potential for CE. Particularly among ethnic and indigenous communities, evidence shows that empowering the community without corresponding changes in the system's infrastructure can compromise the trust and relationship-building purpose of CE initiatives, resulting in poor individual health outcomes (46). The social hierarchy experienced by socially disadvantaged groups remains another significant challenge. For example, the CDC model, which was used to reach socially marginalised groups, received minimal support from the hierarchical health education system in China (64). Similarly, the participatory action model, using non-health workers to deliver the intervention among tribal women in India, has the potential for being unrecognised by the Indian health systems (65). A CBPR-led obesity intervention found that post-intervention, the obesity levels were still higher among black and Hispanic children compared to white or Asian children (69). These results suggest that when CBPR is used in multi-ethnic samples the approach needs to be tailored for each ethnic subgroup. Similarly, Schultz et al. (91) found that CBPR interventions with differing levels of engagement between ethnic subgroups resulted in disproportionate outcomes between ethnic groups due to lack of appropriate programme tailoring. Kim et al. (71) showed that although the lay health advisor model is a sustainable approach, due to the inherently low health literacy levels of the rural lay advisors it compromised the intervention quality, resulting in a short-duration low-intensity programme. Available evidence shows that the lay health advisor model as a primary intervention strategy has limited benefits in achieving health outcomes and has better potential in combination with other health promotion approaches (92).

## Conclusions

We have found that CE improves the health of disadvantaged populations and enhances health programme participation and retention within ethnic minority, indigenous, and immigrant communities who are usually excluded from research and innovative programmes. Despite the social hierarchy that exists among marginalised populations, using a collaborative non-hierarchical approach such as CBPR has shown to be successful in forming partnerships and achieving study outcomes. We have analysed the process indicators of success in each of the CE models used and found that power-sharing, community participation, bicultural CHWs, and collaborative partnerships were key to achieving positive study outcomes.

Although we have attempted to disaggregate the contributions of CE components to health outcomes from those of community development improvements, due to the benefits of engagement seeping into the broader community there is scope for some overlap of these effects. Although CE is useful in reducing health inequalities, it is labour-, cost-, and time-intensive, and its effectiveness varies according to the type of intervention and CE model used. We have found that high-quality CE is often compromised by a lower quality research methodology; in addition, due to several associated methodological challenges, RCTs are not the most effective approach to evaluate CE interventions. Several gaps in the current measurement of CE in heath intervention studies suggest the need for development of innovative frameworks and approaches to demonstrate the effect of CE on health outcomes in a comprehensible rigorous way.

### Policy implications

Our review found positive impacts of CE on the health of disadvantaged populations; however, due to the lack of tools to accurately measure CE, the quantitative relationship between elements of CE and health outcomes could not be determined. In order for researchers to be able to accurately demonstrate the direct impact of CE initiatives, psychometrically robust tools measuring the dimension of CE in existing models are needed. Given that there is no ‘one size fits all’ CE model, health interventionists using CE models should include measurements of CE in addition to other variables in data analysis to demonstrate its relationship to the outcome variables.

## Supplementary Material

Exploring the role of community engagement in improving the health of disadvantaged populations: a systematic review
