# Supplementary material for: Exploring the role of community engagement in improving the health of disadvantaged populations: a systematic review
Source: Glob Health Action. 2015 Dec 18;8:10.3402/gha.v8.29842. doi: 10.3402/gha.v8.29842 (PMC4685976; doi:10.3402/gha.v8.29842)
Supplement: Exploring the role of community engagement in improving the health of disadvantaged populations: a systematic review [file GHA-8-29842-s001.docx]

| **Table 2: Study characteristics of articles included in the systematic review** | | | | | |
| --- | --- | --- | --- | --- | --- |
| **Reference** | **Study population/**  **Country** | **Study design and sample size** | **Type of community engagement** | **Outcome measures** | **Study quality & Limitations** |
| Balcazar et al 2010 | Hispanic community members majority female; mean age =54 yrs  USA | **Experimental:** Randomised Controlled Trial. Sample size: (n=328).Intervention arm (n=192) received a four-month behaviour change program. Control arm (n=136) received usual care | CBPR using CHWs  **Process:** a) community advisory council b) CBPR partnerships between three academic partners, health clinic, existing CHW network organizations and the advisory council.  **Purpose**: a) intervention development and implementation b) ongoing project involvement of CBPR partners | **Primary outcomes:** Health behaviours (salt and fat consumption); BMI; cholesterol, blood pressure  **Secondary outcomes:** Awareness of CVD risk factors | **Study Quality:** Moderate.  Difference in baseline values between groups  **Limitations**: the control group received baseline results which could have resulted in a possible contamination of findings. |
| Baqui et al 2008 | Pregnant women mean age 27 years  Bangladesh | **Experimental:** Cluster three-armed randomised controlled trial. Sample size: (n=24 clusters). The 30-month intervention consisted of 1) CHW-delivered home-care arm (n=8 clusters, 36,059 participants) 2) the community-care arm (n= 8 clusters, 40,159 participants) 3) the control arm (n=8 clusters, 37,598 participants) receiving usual health care. | CHW  **Process:** a) community meetings with pregnant mothers b) engaging husbands in markets and mosques c) advocacy meetings with local elders d) recruitment of community volunteers  **Purpose:** a) intervention development and delivery; b) community surveillance and mobilisation | **Primary outcomes:** Reduction in neonatal mortality  **Secondary outcomes:**  Ante-natal visits from trained providers, iron/ folic-acid supplements, use of clean cord-cutting instruments, tetanus-toxoid immunisation. | **Study Quality:** Good. 85% retention, intention to treat analysis (ITA), no baseline differences between arms  **Limitation**: recall bias as follow-up data collection on antenatal and neonatal practices occurred at 3 years after baseline. Contamination between control and intervention groups due to geographical proximity of clusters. |
| Bender et al 2013 | Low income Hispanic mothers mean age 27 yrs with children mean age 3.6 years  USA | **Quasi-experimental**: Single group pretest–posttest study.  Sample size: (n= 33 mother–child dyads)  A 9 month CHW-led child-hood obesity intervention targeting health behaviour modification | CHW  **Process**: a) Establishment of stakeholder meetings for planning CHW-led intervention; b)Focus groups with community members for identification of program goals; c) individual interviews with community stakeholders for exploration of survey items  **Purpose:** a) to culturally and linguistically adapt the intervention and survey instruments; b) to address health care access barriers; | **Primary outcomes:**  maternal pedometer step count; children’s consumption of sugar-sweetened beverage;  **Secondary outcomes:**  children’ water and milk consumption; maternal and child BMI | **Study Quality**: Moderate. No control group limiting testing of intervention  effectiveness; convenience sampling  **Limitations**: Mid-program adaptations compromising internal validity. Selection bias in recruitment |
| Charania et al 2012 | Community members from three remote First Nations communities in sub-arctic Ontario  Canada | **Qualitative study**  Sample size: (n=44)  Semi-structured interview (n=13), unstructured interviews (n=4) and committee meetings (n=27) were used to implement community engagement | CBPR  **Process**: a) community advisory group involving community health centres and elected community members (Band Council) b) community pandemic committee with 10 representatives from health centre, hospitals, nursing station, Band Council, educational services, clergy, stores, water treatment plant, and emergency medical services  **Purpose:** a) generation of community-specific pandemic plans, following revisions through community engagement process; b) validation and dissemination of findings | **Study outcome:**  Development of culturally-appropriate community-level pandemic plans | **Study quality** – Good. Procedural rigour in sampling, data collection demonstrated; theoretical consistency in findings seen  **Limitations** – Due to unique characteristics of these communities, generalisability of findings is limited |
| Chomitz et al 2010 | African American, Hispanic and Asian children mean age = 7.7 yrs.  USA | **Observational:**  Longitudinal study  Sample size: (n = 1,858)  Studying the impact of the Healthy Living Cambridge Kids, a three-year multicomponent program targeting primary school children’s weight and fitness from baseline (pre-program) to follow-up (3 years) | CBPR  **Process:** a) multidisciplinary coalition comprising parents, teachers, elected officials, public health practitioners and researchers b) collaborative partnerships between coalition, schools, community health centres and public health department c) community forums  **Purpose:** a) needs assessment and formative research, b) developing, pilot testing and implementing the intervention c) dissemination of findings and recommendations to policymakers. | **Primary outcomes:**  BMI z-scores; fitness tests scores | **Study quality**- Poor. Attrition bias - the loss to follow up was 48%  **Limitations** - the lost to follow-up sample differed from the final sample in being Asian, older (9.1 yrs), and lower fitness test score (26.4 vs. 29.9% in final sample), affecting the validity of the results. |
| Chung et al 2009 | African American community members majority female aged 41-50 yrs  USA | **Quasi experimental:** Pre-test post-test study  Sample size: (n=747)  Using questionnaires, the ‘Talking wellness’ program including photo-exhibits (n=747) and spoken word/comedy drama events (n=107) as evaluated for effects on depression. | CPPR  **Process:** a) multidisciplinary coalition involving community health organisation, universities and non-profit research organisations; b) working groups comprising 10 academic members (7 White, 2 Asian American, 1 African American) and 64 ethnic community members.  **Purpose:** a) program and survey instrument development b) coordination of participant recruitment and data collection c) validation of findings. | **Primary outcomes:**  Collective efficacy; community engagement; social stigma  **Secondary outcomes:**  Level of depression knowledge; exposure to Healthy African American Families activities | **Study quality** – Moderate. Convenience sampling; almost 30% missing data at follow-up  **Limitations** – risk of obtaining already ‘engaged’ sample through convenience may have inflated the relationship between event attendance and community engagement |
| Cohen et al 2013 | Parks used by ethnic Los Angeles community  USA | **Experimental:** Randomised controlled three-arm trial  Sample size: (n=50 parks) The 3 arms included 1) park director (PD n=17 parks) arm; 2) PD and park advisory board (PD + PAB, n=16 parks) arm; 3) control (n=17 parks) arm. | CBPR  **Process:** a) community advisory board comprising community members and bilingual community health promoters b) collaborative partnership between recreation and park dept. park staff and advisory board  **Purpose**: a) formative research and park-specific needs assessment b) coordination of marketing strategies; c) development and implementation of the intervention d) interpretation of results | **Primary outcomes:**  Change in the number of park users, change in the level of park-based physical activity  **Secondary outcomes:** Park-based interventions, park activity-related expenditure | **Study quality**- Good.  No baseline differences between arms, 92% retention  **Limitations**- applying CBPR compromised the rigorousness of the RCT as data collectors were not blinded to assessment which may have introduced bias. |
| Collie-Akers et al 2013 | Hispanic community members of the Latino Health for All (LHFA) Coalition  USA | **Qualitative study**  Sample size: (n=40)  Using content analysis of policy documents and self-reporting by community members at committee meetings (n=40) the Latino Coalition CBPR approach was evaluated | CBPR  **Process:** a) community advisory board comprising 10 Latino community members and Latino-based organisations b) collaborative partnership between academics and community members  **Purpose:** a) development of action plans, community mobilisation, implementing community and systems changes b) supervision of evaluation, data analysis and dissemination of results. | **Study outcome:**  Evaluation of the impact of the Latino Coalition on community/system level changes on nutrition, physical activity and access to health screenings | **Study quality** – Moderate. Credible and transferable findings however theoretical connections inadequate.  **Limitations** –Psychometric properties of intensity scoring tool not tested. |
| English et al 2008 | American Indian tribal women (Ramah Band of Navajo Indians) mean age 62 years  USA | **Qualitative study**  Sample size: (n =39)  Focus groups (n=25) and surveys (n =39) used to develop and evaluate a pilot mammography intervention targeting breast cancer early detection | CBPR  **Process:** a) collaborative partnerships involving tribal community board, health dept., and university b) multidisciplinary coalition comprising members from field health, behavioural health, community wellness, health education, finance, and community leadership  **Purpose:** a) formative research and needs assessment b) improve public health knowledge and skills among tribal health providers; c) strengthen local mammography services infrastructure and adopt local policy initiatives to achieve tribal cancer control | **Study outcomes:**  Description of the development and evaluation of a breast cancer screening program; participation rates in the program | **Study quality** – Good. Analytical rigour and theoretical connections demonstrated;  **Limitations-** sample limited to older tribal women restricting generalisability; input from traditional healers, husbands and non-tribal providers who are important stakeholders in women’s health were not collected. |
| Ferrera et al 2015 | Low-income undocumented Latino immigrant youth aged 14-19 yrs USA | **Mixed methods study**  Sample size: (n=23)  In-depth semi-structured interviews (n=23) and closed-ended surveys (n=23) used to evaluate the Youth Health Service Corps, a peer-to-peer health education program targeting youth wellbeing and empowerment | CBPR  **Process:** a) collaborative partnerships involving community-based organizations, staff from CAAAELII (Coalition for African, Arab, Asian, European, Latino Immigrants of Illinois) and new arrival immigrants b) youth advisory board  **Purpose:** a) positive youth development among undocumented youth not eligible for healthcare coverage b) individual and community empowerment c) document the challenges faced by immigrant youth and their families. | **Study outcomes:**  Evaluation of a) the impacts of the YHSC program on youth wellbeing b) CBPR use within the immigrant community | **Study quality**- Moderate. Theoretical perspective and analytical rigour in qualitative component demonstrated; however, quantitative component was not described  **Limitations** –the study recruited youth who participated in the program for 1 year, inflating the results as data was collected from more ’engaged’ youth |
| Fialkowski et al 2014 | Community members from 20 communities in US Affiliated Pacific Islands, Hawaii  and Alaska  USA | **Qualitative study**  Sample size: (n=912)  Community meetings and community feedback meetings (n=912) were used to identify environmental intervention priorities to address behavioural outcomes | Children’s Healthy Living Program Community Engagement Process based on the ANGELO (Analysis Grid for Elements Linked to Obesity) framework  **Process:** a) local advisory committee comprising teachers, parents, cultural and religious leaders, govt. dept. representatives and academics b) key informant interviews with early childhood community agencies’ staff c) community meetings with target groups (parents and teachers)  **Purpose:** a) identification of community’s assets and needs related to healthy eating and active living b) advocacy for CHL-related policy changes c) ensure community alignment of the CHL program d) development of action plans | **Study Outcome:**  Description of a community  engagement process used by the Children’s Healthy  Living (CHL) Program | **Study quality** – Good. Adequate description of the process of data analysis; procedural and analytical rigour demonstrated  **Limitations –** Adherence to the research protocol compromised alignment to community needs. The tailored approach restricts generalisability of findings |
| Gao et al 2007 | Homo-sexual gay men mean age 26 yrs  China | **Experimental**: Non-randomised controlled study.  Sample size: (n=160)  Intervention arm (n=80) received Participatory Entertainment-Education  and control arm (n=80) did not receive any materials. | Culturally appropriate Diffusion Communication model  **Process:** a) Gay-bar based series melodrama and small media events 2) outdoor edutainment diffusion activities  **Purpose:** a) development of a culturally appropriate innovative communication approach to promote behavioural change among marginalized group b) improvement in shared health decision making | **Primary outcomes:** Condom use among MSM (Men who have sex with men) and HIV-related knowledge, attitudes and sex practices  **Secondary outcomes**:  Change in number of sexual partners | **Study quality** –Moderate. Baseline differences between 2 arms found. Non-probabilistic sampling  **Limitations** - Non-random sampling and lack of randomisation affecting external validity of results |
| Goldberg et al 2011 | Latino/African American community partners, program developers/staff  USA | **Mixed methods study**  Sample size: (n=53)  Using surveys (n=35), focus groups (n=27) and interviews (n=24) the adolescent pregnancy prevention program - FOCUS (Families in Our Community United for Success) was evaluated | FOCUS community engagement model  **Process:** a) community coalitions with 53 community partners from the government, educational, financial, not-for profit organisational institutions, and community members; b) community asset-building through staff investment  **Purpose:** a) identification of key strategies that support strong community partnerships b) facilitate program participation and retention | **Study outcome**:  Evaluation of the impact of FOCUS CE model on program implementation, feasibility, sustainability, and teen pregnancy prevention outcomes | **Study quality** – Good. Integration of qualitative and quantitative results demonstrated; data collection informed by theory  **Limitations** – using an ‘engaged’ sample to evaluate the program may have yielded skewed results |
| Greenwald et al 2006 | 2 studies -  1)Five-city study - ethnic youth mean age 13.5 yrs  2) School System study with seventh and ninth grade students  USA | **Observational:** Longitudinal study (Five-city study) Sample size: (n=238). Participants exposed to a youth tobacco program; youth development and tobacco-related attitudes measured at baseline and 6 months post-program. School system study (cross-sectional) Sample size: (n=1315) measured same variables post- program | Youth development model  **Process**: a) community participation among youth in anti-tobacco initiatives; b) partnerships between youth and the broader community through organised activities such as sports clubs, community service, career planning, and support for teen parents  **Purpose**: a) to promote youth wellbeing including personal confidence, independence, empowerment and problem-solving b) to promote social cohesion and social networking | **Primary outcome:**  Statistical independence  of Youth Development Model dimensions; assessment of model dimensions’ relationship with  tobacco-related attitudes and behaviour. | **Study quality** – Poor. Loss to follow up 60%; no information on differences between this group and original sample.  **Limitations-** Attrition bias affecting validity of results. Larger sample in School system study was only used for a cross-sectional analysis. no differences between the 2 study findings |
| Harvey et al 2009 | African American and Latina women  USA | **Mixed methods study**  Sample size: (n=1428)  Using participant observation, group interviews, surveys and written tests the 18- month Healthy Connections (HC) project involving recruiting and training of 8 CHWS was evaluated | CBPR using CHWs  **Process:** a) collaborative partnerships between academia, public health practitioners, and the community b) project steering committee with representatives from five community-based organizations, community members, local health dept., managed care provider and an academic institution c) House Parties - small, informal gatherings of community residents facilitated by CHWs  **Purpose:** a) implementation of the program b) training of CHWs to conduct health screening c) to assess program effects | **Study outcome:**  Evaluation of the Healthy Connections project using a CBPR approach | **Study quality** – Poor. Lack of analytical rigour; process of triangulation unclear.  **Limitations** – evaluation of effectiveness was challenged by the huge loss to follow-up (49%). |
| Kim et al 2008 | Rural Africa American community members 70% female mean age 54 yrs  USA | **Mixed methods study**  **Sample size:** (n=73)  Non-randomised 2 arm trial. Intervention arm (n=36) received an 8-week faith-based weight loss program, control arm (n=37) received the program after the study.  In-depth interviews (n=12) and focus groups (n=35) conducted to evaluate the program | CBPR  **Process:** 1) community coalition comprising pastors, church board representatives, and congregation members of three rural African American churches  **Purpose:** a) formative research to develop the intervention b) development of survey instruments c) ensure program feasibility and sustainability | **Study outcomes:**  Change in weight, waist and hip circumference, physical activity; evaluation of the program | **Study quality** –Good.  Integrated efficacy and interpretive rigour of qualitative and quantitative data demonstrated  **Limitations**- Lack of randomisation and purposive sampling affecting the validity and generalisability of the results |
| Napoles et al 2015 | Spanish-speaking Latinas with breast cancer mean age = 50 years  USA | **Experimental:** Randomised controlled trial  Sample size: (n=151)  The intervention arm (n=76) received an 8- week peer-delivered cognitive–behavioural stress management program. The control arm (n=75) received the program after the study | CBPR using peer-facilitators  **Process:** a) community partnerships involving 10 community-based organisations and 6 clinical sites, b) community advisory board comprising Latina cancer survivors, advocates, oncologists, and social service providers, c) steering committee involving investigators and study field staff.  **Purpose:** a) formative research to culturally adapt the program b) implementation of the Spanish-adapted program c) recruitment and training of peer-facilitators | **Primary outcomes:** Breast cancer–specific quality of life; breast cancer-specific distress; general distress | **Study quality**- Good. ITA, 95% retention, no baseline differences between arms  **Limitations** –Control participants were allowed to access other cancer services, affecting the accuracy of the results. |
| Parker et al 2008 | Low income African American/ Hispanic community  USA | **Experimental:** Two arm RCT  Sample size: (n=298 households)  Intervention arm (n=116) received 12 month CHW-led asthma control intervention while control arm (n=111) received basic information on asthma | CBPR using CHWs  **Process**: a) steering committee with representatives from eight community-based organizations, one health service agency, one state agency, one academic institution, and a community member  **Purpose:** a) development of survey instruments and intervention b) recruitment and training of CHWs d) dissemination of findings in culturally appropriate way | **Primary outcomes:** Asthma symptoms; lung function tests; asthma medication use; asthma-related medical visits  **Secondary outcomes:**  caregiver mental health; environmental trigger-reducing behaviour | **Study Quality** –Good. ITA, no baseline differences between arms, 90% retention  **Limitations** – the single annual time-point for post-intervention measurement precludes detection of minor symptom differences and raises the possibility of recall bias |
| Pazoki et al 2007 | Iranian women mean age 39 years  Iran | **Experimental:** Randomised controlled trial  Sample size: (n=335)  Intervention arm (n=170) received an 8-week lifestyle modification program, and control arm (n=165) did not receive any program | CBPR using CHWs  **Process**: a) community advisory board consisting of members of Bushehr Province Women Commission, local NGOs, and representatives of three community-based organizations b) twelve forums among community members, academic researchers, health care providers, and policy-makers  **Purpose:** a) needs assessment b) development and cultural adaptation of program materials c) program implementation d) training CHWs based on ‘train the trainer’ model e) capacity building of CHWs | **Primary outcomes:** moderate intensity physical activity  (>=30 min/day, 5 days /wk) and vigorous intensity physical activity (> =20 min/day, 3 days/wk);  **Secondary outcomes:**  fasting blood sugar, cholesterol, blood pressure , BMI, waist circumference, healthy heart knowledge, and awareness. | **Study quality**- Good. >90% retention, no baseline differences between groups  **Limitations** – study solely relied on self-reported physical activity when validation of findings was not possible. |
| Phillips et al 2014 | 40 deprived neighbour-hoods in London  UK | **Experimental:** Cluster Randomised Controlled trial  Sample size: (n= 3986)  Intervention arm (n=20 neighbourhoods) received ‘Well London’, program promoting healthy eating, physical activity and mental wellbeing, the control arm (n=20 neighbourhoods) did not receive any program. | The Well London Community Engagement approach  **Process:** a) community advocacy on utilising food co-ops and green urban spaces b) community-based healthy cooking classes, physical activity sessions and mental health promotion c) volunteer-organised community events and community gardens  **Purpose:** a) to create a health enabling environment b) to develop health knowledge, new behaviour strategies, social networks, self-efficacy and skills among participants, c) to achieve community empowerment. | **Primary outcomes:** Healthy eating; physical activity;  General Health Questionnaire -12 score;Warwick–Edinburgh Mental Well-being Scale score  **Secondary outcomes:**  Unhealthy eating score: perception of neighbourhood cohesiveness | **Study quality**- Good. No baseline differences between arms, 96% retention, ITA  **Limitations** -low participation rates (28%) and sampling bias; high migration rates in intervention neighbourhoods  (40% of follow-up respondents moved)  affecting the internal validity of results |
| Tripathy et al 2010 | Rural, Low income Indigenous women aged 15–49 years  India | **Experimental**: Cluster randomised controlled trial.  Sample size: (n=36 clusters) Intervention arm (n=18 clusters) received monthly peer-delivered intervention for 20 months. Control arm (n=18 clusters) received usual care. | Participatory action cycle using peer- facilitators  **Process:** a) cluster-level health committees with ten village representatives b) workshops with frontline government health staff c) monthly meetings with community partners  **Purpose:** a) training community partners in participatory techniques; b) obtaining community members’ feedback on local health services c) training peer facilitators in intervention delivery | **Primary outcomes:**  Neonatal mortality reduction; maternal depression scores  **Secondary outcomes:**  stillbirths, maternal and perinatal deaths; uptake of antenatal and delivery services;  delivery and post-delivery care | **Study quality**- Good. >90% retention, no baseline differences between groups, ITA  **Limitation** – lack of blinding in group allocation and inter-cluster migration may have affected the results. |
| Undie et al 2014 | Mothers aged 30–32 years in Naivasha District, Rift Valley  Province  Kenya, Africa | **Experimental:** Cluster randomised controlled trial  Sample size: (n=559; 6 communities)  Intervention arm (N=3 clusters; n=421) received the Community Mobilization for Post-abortion Care program, control arm (N=3 clusters, n=200) received the program after the study | Community action cycle  **Process:** a) participatory capacity-building process for CHWs involving community mentoring and mobilization b) community–facility linkage meetings with trained CHWs c) capacity-building sessions for clinical and family planning staff  **Purpose:** a) needs assessment on barriers to use of family planning and post-abortion care services b) development of participant education materials for home outreach visits | **Primary outcomes**:  Awareness of family planning and early pregnancy bleeding; family planning and post-abortion care service utilisation. | **Study quality** – Poor. Unequal sample size as intervention arm had twice as many participants as control; poor adjustment of baseline characteristics when assessing intervention effects  **Limitations** - difference in baseline characteristics between 2 arms found, cluster matching characteristics were not reported, affecting the internal validity of results |
| Wells et al 2013 | Adults with depressive symptoms 85% Latino/African American background mean age = 46 yrs  USA | **Experimental:** Cluster, Randomized, Comparative-Effectiveness Trial  Sample size: (n=1018)  The 2 study arms included 1) community engagement and planning arm; (n=514) 2) Resources for services arm based on expert assistance for individual programs. (n=504) | Community-Partnered Participatory Research (CPPR)  **Process:** a) steering council comprising academic institution, community health organisation, NGOs, health services, community health and faith partnership b) workgroups and community forums under leadership of the steering council  **Purpose**: a) project planning and budget allocation b) development of culturally competent practices in project implementation and adaptation of depression care toolkits for ethnic communities | **Primary outcomes:**  Mental health-related quality of life (MHRQOL); health service utilisation  **Secondary outcomes:**  Homelessness risk factors; mental wellness; resilience; physical activity; employment | **Study quality** –Good. 75% retention, ITA, baseline characteristics between groups found to be similar  **Limitation** –the study did not have a usual-care arm, but compared two active interventions |
| Wright et al 1997 | Navajo mothers mean age 27 years  USA | **Mixed methods study**  Sample size: (n=250)  Questionnaires (n=250) and ethnographic interviews (n=35) were used to evaluate the breastfeeding program | Community Empowerment model  **Process:** a) collaborations between govt. health service, community health service and tribal community consultants b) partnership with tribal foster grandparent program c) 3-day conference on health education  **Purpose:** a) formative research and needs assessment on tribal barriers to breast feeding, b) co-development and cultural adaptation of program | **Study outcomes**:  Change in duration of exclusive breastfeeding | **Study quality** – Good. Qualitative component informed the quantitative, both theory-based; interpretive rigour demonstrated  **Limitations** –Empowerment effects on the community were not studied. |

| **Table 3: Synthesis of results** | | | | | | | |
| --- | --- | --- | --- | --- | --- | --- | --- |
| **Reference** | **CE Model** | **Study outcome findings** | **Key CE component/s achieving study success*** | **Level of CE^#^** | **Extent of CE in research ^** | **Evaluation of CE** | **Type of CE Evaluation and findings** |
| Balcazar et al 2010 | CBPR | Improvement in self-reported behaviours - weight control practices, (Int. vs Control, mean (SD) 2.0 (0.6) vs 1.9 (0.6),p=0.01) salt (2.0 (0.5) vs1.8 (0.5), p<.001), cholesterol and fat intake (1.9 (0.7) vs1.7 (0.6),p=.01), no BMI changes. | **Context**: Perceived severity of health issues  **Group dynamics**:  ***Structural:*** diversity/formal agreements  ***Relational***: Participatory decision-making and negotiation  ***Individual:*** Bridge people on research team  **Intervention** : bidirectional translation, implementation and dissemination  **Outcomes**: cultural renewal, reduced health disparities; empowerment | Moderate | Good | **✓** | **Process evaluation**  **Positive** – community partners valued the handbook on CBPR principles and project activities; bilingual newsletters to broad community improved recruitment strategies and helped disseminate baseline results which improved health behaviours among the community  **Negative** - integrating CBPR with research was time and labour-intensive, tensions between ‘rigid’ RCT and ‘flexible’ CBPR needs |
| Baqui et al 2008 | Community Health Worker model | Reduction in neonatal mortality (adjusted relative risk 0·66; 95% CI 0·47–0·93, p=0.01) in home-care arm | Promoting health message through religious discourses at mosques; using traditional birth attendants as community mobilisers | Moderate | Poor | X | None |
| Bender et al 2013 | CHW | Improved maternal physical activity in step counts, (4302; 95%CI: 1650, 6953, p<.01); reduced children’s consumption of sugary drinks (ounces per day) (- 3.0; 95%CI: - 6.9, - 2.5) p<.01) | Cultural adaptation of the program by exploring superficial and deep cultural perceptions; Program alignment with patriarchal traditions; Incorporating terms and slang commonly used by low income Hispanics into program;  Incorporation of culture-friendly message on diabetes risk | Moderate | Good | **✓** | **Formative, process and outcome evaluations**  **Positive-** cultural adaptation of the program resulted in 77% program completion rate; high program satisfaction, approval of translated surveys, requests for more similar programs, satisfaction with child care provision; establishment of informal social meetings, post-program sustainability  **Negative** – repeated program modifications to fulfil community needs compromised the rigour of the study |
| Charania et al 2012 | CBPR | Culturally appropriate locally relevant community-developed pandemic plans with information on surveillance, health services, supplies, drugs, list of infection control protocols | **Context:** perceived severity of health issues  **Group dynamics**:  ***Structural***: Real power sharing  ***Relational:*** Integration of local beliefs to group process  ***Individual:*** cultural identity  **Intervention** : Intervention informed by local settings and organisations  **Outcome:-**change in policy; empowerment | Moderate | Good | X | None |
| Chomitz et al 2010 | CBPR | Decrease in mean BMI *z*-scores (−0.04,p < 0.001), obesity prevalence (-2.2%; p < 0.05) and increase in fitness of 14.6% (p<.001) | **Context:** historic degree of collaboration & trust between leadership and community  **Group dynamics:**  ***Structural:*** length of time in partnership  ***Relational***: participatory decision-making and negotiation  ***Individual:*** cultural identity  **Intervention** : Research and evaluation design reflects partnership input  **Outcomes**: change in policy; improved health disparities; empowerment | Good | Good | X | None |
| Chung et al 2009 | CPPR | Collective efficacy independently predicted community engagement (B=0.64–0.97; p<.001).in improving depression care | **Context** : perceived severity of health issues  **Group dynamics:**  ***Structural:*** Real power sharing  ***Relational:*** leadership and stewardship from community  ***Individual:*** motivation to participate  **Intervention :** Shared learning between academic and community knowledge  **Outcomes**: culture revitalisation; empowerment | Good | Good | X | None |
| Cohen et al 2013 | CBPR | Increase in park use -600 more visits/week/park, and 1830 more MET-hours of physical activity/week/park p<.05 in intervention compared to control group. | **Context** : environmental factors  **Group dynamics:**  ***Structural:*** Formal agreements  ***Relational:*** Task roles and communication  ***Individual:*** Community reputation of Partner investigator  **Intervention :** informed by local setting and organisations  **Outcomes:** change in policy; transforming environmental conditions | Moderate | Good | **✓** | **Process evaluation**  **Positive** - Marketing improved park use, the training facilitated peer support, scalability achieved  **Negative** – Irregular meeting attendance, lack of combined vision in goal achievement between park staff and park volunteers, trade-off between tailoring and standardising in multi-site study (Derose 2014) |
| Collie-Akers et al 2013 | CBPR | Ongoing initiatives: gardens (low reach, high strategy, intensity score 2.1) conversion of park into soccer field (high reach, high strategy, intensity score 3.0) and health-related translation services (medium reach, medium strategy, intensity score 2.1). | **Context** : national and local policy trends  **Group dynamics:**  ***Structural:*** formal agreements  ***Relational:*** Task roles and communication  ***Individual***: motivation for participating  **Intervention** : bidirectional translation, implementation and dissemination  **Outcomes:-** change in policy; transforming environmental conditions | Good | Good | **✓** | **Outcome evaluation**  **Positive** - increased accountability of stakeholders, identification of environmental changes influencing health outcomes, transparency in the actual progress of programs documentation  **Negative** – The monitoring system showed only a quantification of outcomes without outlining the study-related CBPR challenges |
| English et al 2008 | CBPR | Improved participation (77%) in mammography programs; 36% received their first mammogram; 100% of participants favoured ongoing participation and recommending the program to family and friends. | **Context** : cultural/perceived severity of health issues  **Group dynamics:**  ***Structural:*** real power/resource sharing  ***Relational:*** Integration of local beliefs to group process  ***Individual:*** cultural identity  **Intervention** : intervention adapted and created within local culture  **Outcomes:-** change in policy; reducing health disparities; empowerment | Good | Good | **✓** | **Process evaluation**  **Positive**- participants valued the program lunch (70%), transportation (40%), social support (35%), health education (35%), and friendly staff (35%), and face-to-face home recruiting; involvement of tribal agency (a trusted source) and self-determination of the tribe to improve their health  **Negative**- negative cultural perceptions of cancer and fear of talking about cancer(22%), inadequate local mammography services and infrastructure |
| Ferrera et al 2015 | CBPR | Improved individual and community empowerment levels; 100% program participation and positive program experience | **Context** : cultural  **Group dynamics:**  ***Structural:*** real power/resource sharing  ***Relational:*** Integration of local beliefs to group process  ***Individual:*** motivation for participating  **Intervention** : intervention adapted and created within local culture  **Outcomes:-** change in policy/practice; reducing health disparities | Good | Good | **✓** | **Process evaluation**  **Positive** – non-hierarchical approach and mentorship from program coordinator sustained program engagement; undocumented and non-health insured youth migrants felt empowered to be included in research protocol development; improved participation from youth in leadership and advocacy activities; 23 youth provided health education to 800 community members  **Negative** – Immigration status causing fear and reluctance to participate in research study, difficulty in obtaining parental consent due to cultural barriers |
| Fialkowski et al 2014 | ANG-ELO frame-work | Community-identified environmental priorities include; role modelling;  enhancing access to healthy food, clean water and physical activity venues; and healthy living education to community | **Local advisory committee:** advise on partnership initiation; advocacy of project-initiated policy change and sustainability  **Key informant meetings:** development of shared vision with community involvement;  **Target community meetings**: community input into intervention design; adaptation based on community needs  **Community partnerships:** guidance on alignment of project with community needs  **Community Feedback meetings:** Community-initiated development of action plans; discussion on implementation | Moderate | Moderate | **X** | **None** |
| Gao et al 2007 | CDC model | Condom use increased by 67.4% (p<.001) for vaginal sex, 72.5% (p<.001) for anal sex, and 14.5% (p<.01) for oral sex; HIV/AIDS-related knowledge increased by 71.2% (p<.001) | **Focus on individual socio-psychological perspective:** Increased awareness to  promote attitudes and behavioural change  **Focus on social groups and networks:** People-centred participatory and diffusion  communication approach  **Focus on social environmental**  **change and culturally appropriate approach**: Community support and  improving culturally appropriate contexts | Good | Good | **X** | **None** |
| Goldberg et al 2011 | FOC-US model | 22% increase in projected program recruitment; 88% retention; 71% of participants had developed new community connections; 93% success in creating community interests; increased awareness of teen pregnancy as an issue; community volunteering (>50%) | **Core principle I: promote asset-building/recognize strengths**: staff investment, marketing strategies to enable empowerment of community;  **Core principle II: build a shared vision, and respect individual and organizational interests**: establish dialogue with community; develop tailored messages  **Core principle III: be inclusive and value equity:** power sharing, social networking  **Core principle IV: value informality and flexibility:** organic strategies such as regular informal community gatherings | Moderate | Moderate | **✓** | **Process evaluation**  **Positive-** program was successful in developing commitment to participate (96%) ; in expanding the community partners’ organizational capacity (78%). Positive parental feedback on youth behaviours, enhanced social connections and support, improved knowledge on community resources, improved communication between parents, adolescents and teachers  **Negative** – lack of evidence of program reducing teen birth rates, lack of adequate adolescent health services and lack of clear guidelines on the role of partner organisations’ contributions |
| Greenwald et al 2006 | Youth development model | No statistically significant relationships between all 3 youth development variables (personal development, social development and engagement) and tobacco-related attitudes and behaviour | **Personal development** : a sense of self-worth, independence and control over one’s life, competence and problem-solving  **Social development**: sense of belonging, group membership, and close ties with friends and family members  **Community engagement :** participation in community activities, boredom | Poor | Poor | X | None |
| Harvey et al 2009 | CBPR | Reach exceeded the estimated target by 40%; 70% of screened participants had blood pressure readings above normal. | **Context** : perceived severity of health issues  **Group dynamics:**  ***Structural***: length of time in partnership  ***Relational***: Leadership and stewardship  ***Individual:*** motivation for participating  **Intervention** : research and evaluation design reflects partnership input  **Outcomes:-** change in screening practice; reducing health disparities | Moderate | Good | **✓** | **Formative, process and outcome evaluation**  **Positive**- 55% indicated that they had not been previously diagnosed with high blood pressure. 51% of hypertensive participants were given referrals to local low cost health providers; 100% of CHWs completed health worker training and 88% completed social network training; improved problem-solving skills and information sharing among health workers  **Negative**- poor retention of bilingual staff related to stipend cuts, poor data collection systems for participant follow-up and social networking component of the program |
| Kim et al 2008 | CBPR | Improvement in weight mean (SE) (–2.8 (0.003) pounds, p<.01), hip circumference (–2.3 (1.2) cm, p=.05) physical activity in metabolic equivalent task (6.4 (2.2) METs, p=.01) | **Context** : perceived severity of health issues  **Group dynamics:**  ***Structural:*** Real power-sharing  ***Relational***: Participatory decision-making and negotiation  ***Individual***: individual beliefs, spirituality and meaning  **Intervention** : intervention created within local culture  **Outcomes:-** culturally –based sustainable intervention; empowerment | Moderate | Good | **✓** | **Process evaluation**  **Positive**- improved program feasibility, sustainability, reach and effectiveness; greater community cohesiveness and willingness from community organisations to promote health, empowerment of lay health advisors  **Negative**- Lay health advisors had difficulty in coping with reading materials and suggested less text, more hands-on activities, and more examples concerning application of materials |
| Napoles et al 2015 | CBPR | Emotional well-being (quality of life) group mean higher in intervention than the control group, (16.39 vs 14.89; p= .018); group mean on intrusive thoughts lower in intervention group than the control group, indicating less distress (3.87 vs 6.27; p= .046). | **Context** : cultural  **Group dynamics:**  ***Structural:*** real power-sharing  ***Relational***: dialogue, listening and mutual learning; integration of local beliefs to group process  ***Individual:*** bridge people on research team  **Intervention** : Intervention created within local culture  **Outcomes:-** culturally-based and sustainable intervention; reducing health disparities; empowerment-capacity of advisory council | Moderate | Good | **✓** | **Process evaluation**  **Positive**- Using bilingual health workers helped achieve project credibility, and improved recruitment, retention and cultural appropriateness of the program, ongoing communication with community partners facilitated randomisation and helped overcome resistance to participation  **Negative**- due to low medical staffing establishing relationships between clinical staff and community partners was difficult and participant recruitment was delayed |
| Parker et al 2008 | CBPR | Improvement in lung function daily nadir FEV_1_ –forced expiratory volume at one second as % (10.0; 95% CI 0.9, 19.1, p=0.03) and daily nadir PF –peak flow as % (8.2; 95%CI 1.1, 15.2, p=0.02) | **Context** : perceived severity of health issues  **Group dynamics:**  ***Structural:*** real power-sharing  ***Relational:*** participatory decision making and negotiation  ***Individual:*** bridge people on research team; cultural identity  **Intervention** : shared learning between academic and community knowledge; research and evaluation design reflects partnership input  **Outcomes:-** culturally-based and sustainable intervention; reducing health disparities | Moderate | Good | **X** | None |
| Pazoki et al 2007 | CBPR | Increased minutes of physical activity per week mean(SE) (139.81 (23.35) in intervention compared to 40.14 (12.65) (p<.0001) in control group | **Context** : perceived severity of health issues  **Group dynamics:**  ***Structural:*** diversity  ***Relational***: integration of local beliefs to group process  ***Individual:*** cultural identity  **Intervention** : intervention developed within local culture; research and evaluation design reflects partnership input  **Outcomes:-** change in practice; reducing health disparities; | Moderate | Good | **x** | None |
| Philips et al 2014 | Well London model | No impact of the intervention on healthy eating (RR: 1.04, 95% CI 0.93 to 1.17); physical activity (RR:1.01, 95% CI 0.88 to 1.16); mental health and wellbeing measured by GHQ 12 (RR:1.15, 95% CI 0.84 to 1.61) and WEMWBS (mean difference: −1.52, 95% CI −3.93 to 0.88). | **Health enabling environment**: safe neighbourhoods, access to parks, cohesive environmental planning  **Health knowledge and new behavioural strategies**: walking groups, gardening activities, improved knowledge of cooking  **Social networks, support and integration**: mental wellbeing impact assessment, improved social cohesion  **Self-efficacy and skills:** sense of autonomy from volunteering, individual mental wellbeing, community involvement resulting in feeling of usefulness | Poor | Poor | **✓** | **Process evaluation**  **Positive**- the few participants who experienced social cohesion and collective agency experienced positive health benefits  **Negative** - discrepancy in the evaluation of engagement as ‘geographical’ communities in which effects were measured did not correspond with the ‘engaged’ communities; the model was a mixture of CE and health improvement strategies where precise impact of various components on the outcomes was not determined (Derges et al 2014) |
| Tripathy et al 2010 | Participatory action cycle | Neonatal mortality rate was 32% lower in intervention clusters (OR: 0·68, 95% CI 0·59–0·78, p<0.05) compared to control clusters after adjustment | **Phase 1- Identification and prioritisation of difficulties:** Maternal issues, neonatal issues, current practices  **Phase 2: Planning strategies:** identification of barriers to access; identify and prioritise strategies; establish process for information sharing  **Phase 3: Implementing strategies**: establishing protocols; monitoring progress against timelines; addressing obstacles  **Phase 4:Assessment of effect**: dissemination of results to the community; qualitative evaluation of intervention | Moderate | Moderate | **x** | None |
| Undie et al 2014 | Community action cycle | No significant change in increased awareness of early pregnancy bleeding or improved family planning service utilisation | **Organise the community for action**: community mobilisation, involvement and mentoring  **Exploring the health issues and identifying priorities**: raise community health awareness; facilitate self-diagnosis; identify barriers to health service use  **Plan together**: community health communication strategies; resource and capacity of health services assessment  **Act together:** jointly resolving problems; leverage community resources; utilise community-endorsed leadership  **Evaluate together**: health service utilisation; knowledge levels of health issue and existing resources  **Prepare to scale up**: based on resource availability | Poor | Moderate | **X** | None |
| Wells et al 2013 | CPPR | Improvement in mental HRQOL adjusted OR=0.74 (95%CI: 0.57, 0.95), p<.05; improved health service utilisation adjusted OR=0.51 (95%CI: 0.28, 0.95) p<.05; decreased risk of homelessness adjusted OR=0.61, (95%CI: 0.38, 0.96, p<.05) | **Context** : perceived severity of health issues  **Group dynamics:**  ***Structural:*** formal agreements  ***Relational:*** dialogue, listening and mutual learning  ***Individual:*** cultural identity  **Intervention** : shared learning between academic and community knowledge; research and evaluation design reflects partnership input  **Outcomes:-** change in practice; reducing health disparities | Moderate | Good | **X** | None |
| Wright et al 1997 | Community empowerment | Improvement in breastfeeding rates pre- to post-intervention 64 % to 78%, p<.001; decrease in formula feeding rates 85% to 45%,p<.001; increase in breast-feeding initiation 71% to 81%,p<.0001; delay in starting formula feeds (mean age in days: 12 to 48.5,p<.001); increased duration of breast-feeding (mean age in days: 101 to 132, p<.001) | **Gaining entry into the community:** community leaders; academic institutions and volunteer organisations  **Identifying issues of concern to the community:** community’s ‘buy-in’; trust- building between all involved partners  **Prioritizing identified issues:** discussions on community concerns; prioritising topics based on community’s needs  **Formulating a strategy to address the prioritised issue:** participatory group process **Developing and implementing an action plan to resolve the prioritised issue**: community ownership; transfer of leadership to community identified leaders | Moderate | Good | **✓** | **Formative, process and outcome evaluation**  **Positive**- tribal foster grandparent program identified traditional benefits of breast feeding, input from cultural community stakeholders in program design improved the cultural acceptability of program, social marketing strategies improved breastfeeding; increased breast-feeding knowledge levels among fathers and local healthcare providers post-program  **Negative**- time-consuming, community engagement process needed repeated reinforcing, inadequacies in infrastructure impede program sustainability; community empowerment was not measured |

* **CBPR logic model aligned with 9 CBPR principles (P1-P9)**

Context:  community as a unit (P1) and community resources (P2)

Group dynamics: equal partnerships (P3) and co-learning (P4)

Intervention: balance between research and action (P5), program development (P7), dissemination (P8)

Outcome: sustainability (P9)

# **5 levels of CE along a continuum**

1. Inform – providing the community with information on the program
2. Consult- listening to community feedback, not allowing new ideas
3. Involve – allow joint decision-making
4. Collaborate – forming a partnership to carry out the decisions
5. Empower – place final decision-making in hands of the community

Scoring – Levels1-2=poor, levels 3-4=moderate; levels 5 –good; level 6 – excellent

^**Extent of Community involvement in research**:

1. Identification of issues of greatest importance
2. In designing study and development of program
3. Recruitment of participants and retention
4. Development of instrument and pilot testing in same population
5. Implementation of intervention
6. Analysis and translation of findings, dissemination to community

Scoring <=2 criteria – poor; 3-4 criteria =moderate;>5 criteria=good
